# Effects of vildagliptin compared with glibenclamide on glucose variability after a submaximal exercise test in patients with type 2 diabetes: study protocol for a randomized controlled trial, DIABEX VILDA

**DOI:** 10.1186/1745-6215-15-424

**Published:** 2014-11-04

**Authors:** Aline Fofonka, Jorge Pinto Ribeiro, Karina Rabello Casali, Beatriz D Schaan

**Affiliations:** Exercise Pathophysiology Research Laboratory, Hospital de Clínicas de Porto Alegre, Porto Alegre, Brazil; Cardiology and Endocrinology Divisions, Hospital de Clínicas de Porto Alegre, Porto Alegre, Brazil; Department of Science and Technology, Science and Technology Institute, Universidade Federal de São Paulo, São José dos Campos, Sao Paulo, Brazil; Department of Internal Medicine, Faculty of Medicine, Universidade Federal do Rio Grande do Sul, Porto Alegre, Brazil

**Keywords:** Diabetes mellitus, Hypoglycemic agents, Exercise

## Abstract

**Background:**

Cardiovascular disease, endothelial dysfunction, and oxidative stress are common complications among patients with type 2 diabetes (T2DM). In addition to the average blood glucose concentration, glycemic variability may be an important factor for the development of chronic diabetes complications. Patients with T2DM are treated with various types of oral glucose-lowering drugs. Exercise is considered to benefit the health of both healthy and unhealthy individuals, which has been confirmed by a number of scientific research studies in which the participants’ health improved. Our general aim in this study will be to evaluate glucose variability after submaximal exercise test in patients receiving treatment with either vildagliptin or glibenclamide. The specific aims of this study are to evaluate the oxidative stress, endothelial function, and metabolic and cardiovascular responses to exercise under treatment with vildagliptin or glibenclamide. All these responses are important in patients with T2DM.

**Methods/Design:**

This study is a PROBE (Prospective, Randomized, Open-label, Blinded-Endpoint) design clinical trial.

The estimated sample needed is 20 patients with T2DM. In addition to the routine treatment (metformin), patients will receive a second drug orally for 12 weeks: the METV group will receive metformin plus vildagliptin (50 mg twice daily), and the METG group will receive metformin plus glibenclamide (5 to 10 mg twice daily.). Before and after intervention, evaluation of glycemic variability, endothelial function, oxidative stress, and metabolic and cardiovascular response will be performed at rest, during and after a submaximal exercise test (30 minutes, with an intensity based at 10% under the heart rate at the second threshold).

**Discussion:**

In addition to drug treatment, exercise is recommended for treatment of glycemic control in patients with T2DM, especially for its beneficial effects on blood glucose and HbA1c. Few studies have determined the effects of the association between exercise and oral glucose-lowering drugs. The study will be conducted to assess the metabolic and cardiovascular responses at rest, and during and after submaximal exercise in patients receiving one of two oral glucose-lowering drugs (vildagliptin or glibenclamide).

**Trial registration:**

ClinicalTrials.gov Identifier: NCT01867502 study release date: May-17-2013.

## Background

Cardiovascular disease is the main cause of mortality among people with type 2 diabetes mellitus (T2DM) [1, 2]. Accelerated atherosclerosis in these patients is preceded by endothelial dysfunction, inflammatory burden, and increased lipid peroxidation, all leading to enhanced macrophage foam cell formation [2]. In addition to the average blood glucose concentration, acute glycemic fluctuations from peaks to nadirs (glucose variability) may be involved in the development of diabetic complications [3], as they contribute to the generation of excessive protein glycation and oxidative stress [4]. High glucose variability has been shown to be associated with endothelial dysfunction in patients with T2DM and optimal metabolic control [5]. Currently, the mean amplitude of glycemic excursion (MAGE) is one of the most used methods for detecting significant swings in glycemia [6], but other tools may be useful to identify disturbances in glucose variability [7].

Some current treatments for T2DM have already been tested concerning their possible effects in reducing glucose variability as well as reducing glycated hemoglobin (HbA1c) [8]. Exercise, which is one of the cornerstones of treatment for hyperglycemia in T2DM because of its beneficial effect on HbA1c [9], was recently shown to reduce glucose variability in addition to its acute effects on reducing glucose levels [7]. Vildagliptin and sitagliptin are two drugs that were recently evaluated in a study focusing on possible differences in daily glucose fluctuations in patients with T2DM inadequately controlled with metformin, and it showed that vildagliptin was more effective in flattening acute glucose fluctuations over a day [8]. Moreover, acarbose was superior to glibenclamide in reducing MAGE. Therefore, aside from their absolute glucose-lowering effect, it is evident that other effects of different anti-diabetic agents might also be different [10].

The present study will be conducted to test the hypothesis that in combination with metformin, vildagliptin may produce a greater improvement in glucose variability after a submaximal exercise test, compared with glibenclamide. Our general aim will be to evaluate glucose variability after the submaximal exercise test in patients receiving treatment with vildagliptin or glibenclamide. The specific aims of this study are to evaluate the oxidative stress, endothelial function, and metabolic and cardiovascular responses to exercise in patients treated with vildagliptin or glibenclamide.

## Methods/Design

### Research design

This study is a PROBE (Prospective, Randomized, Open-label, Blinded-Endpoint) design clinical trial.

### Outcomes

The primary outcome will be glucose variability evaluation, as evaluated by conventional and non-conventional methods. Secondary outcomes will include oxidative stress, endothelial function, and metabolic and cardiovascular responses to exercise. Table 1 shows the measures that will be evaluate to reach the outcomes.

**Table 1:** **Measures evaluated in order to reach the outcomes**

Time point	Data collection	Measure	Outcomes
At rest	Continuous glucose monitoring system	Glucose values	Glucose variability
	Vascular Doppler ultrasound	Flow-mediated dilatation	Endothelial function
	Blood sampling	Glucagon, glucose, HbA1c, insulin and GLP-1	Metabolic responses
	Impedance cardiography and electrocardiogram	Cardiac output, heart rate	Cardiovascular response
	24 hour urine	F2 isoprostane 8-isoprostaglandin F2α/ creatinine	Oxidative stress
During submaximal exercise tests	Continuous glucose monitoring system	Glucose values	Glucose variability
	Impedance cardiography and electrocardiogram	^a^Cardiac output, heart rate	Cardiovascular response to exercise
	Blood sampling	^a^Glucagon, glucose, insulin, and GLP-1	Metabolic responses
After submaximal exercise tests	Continuous glucose monitoring system	Glucose values	Glucose variability
	Impedance cardiography and electrocardiogram	^ab^Cardiac output, heart rate	Cardiovascular response to exercise
	Ambulatory blood pressure monitoring	24 hour blood pressure variability	
	Blood sampling	^ab^Glucagon, glucose, insulin and GLP-1	Metabolic responses

GLP-1, glucagon-like peptide 1; HbA1c, glycated hemoglobin. ^a^0, 15, and 30 minutes; ^ab^60 minutes after exercise.

### Sample size calculation

According to data reported by Marfella *et al*. [8], a total sample size of 20 patients (allowing for a dropout rate of 10%) should allow detection of a difference between groups with MAGE levels (mean ± SD) of 25.0 ± 16 mg/dl at week 12, assuming statistical power of 90%, and a significance level of 1% (two-sided, two-sample *t*-test).

### Inclusion and exclusion criteria

The inclusion criteria will be age older than 18 years, presence of T2DM, use of metformin, recent HbA1c between 7.5% and 10%, and no involvement in regular physical activity.

The exclusion criteria will be: current smoking; body mass index (BMI) >40 kg/m^2^; presence of proliferative diabetic retinopathy, ischemic heart disease, peripheral vascular disease, cognitive decline or dementia, neurological events, severe depression, or current diagnosed cancer; lactose intolerance; hepatic enzyme levels threefold higher than the reference values; glomerular filtration rate lower than 60 ml/min; blood pressure (BP) over 180/100 mmHg at rest; use of analgesic or anti-inflammatory drugs during the week of the study; use of insulin; and untreated thyroid dysfunction.

### Eligibility assessment and follow-up visits

Eligibility and exclusion criteria will take place in two visits, and will be assessed by medical records, patient interviews and laboratory tests.

A physician will be responsible for the cardiovascular assessment, using the American Heart Association guidelines [11]. The same physician will assess the presence of peripheral vascular disease using the ankle brachial index [12].

Two follow-up consultations will take place after 4 and 8 weeks of treatment to measure BP, heart rate, and body weight.

### Randomization

The randomization sequence will be generated by the R software (v2.12.1 Vienna, Austria, 2011), with a block size of five. Randomization to the metformin plus vildagliptin group (METV) or the metformin plus glibenclamide group (METG) will be performed by a researcher responsible only for this task, who will not participate in the recruitment, assessment, or intervention phases of the study.

### Data collection

Eligible patients will initially perform a maximum effort test, and 48 hours later, they will begin the study protocol, as follows:

- Day 1: Begin 24-hour urinary collection, perform vascular Doppler ultrasound to evaluate endothelial function, and then insert glucose sensor subcutaneously to begin continuous glucose monitoring system (CGMS) evaluation.
- Day 2: End the 24-hour urinary collection, carry out the submaximal exercise test (with blood collection at baseline, 15, and 30 minutes into the session, and 60 minutes after recovery). Assess heart rate variability 10 minutes before and after the submaximal exercise test, and begin 24 h ambulatory blood pressure monitoring (ABPM).
- Day 3: Removal of the glucose sensor; end 24 h ABPM. Randomization.

This same protocol will be repeated at the end of the 12-week treatment with vildagliptin or glibenclamide.

**Figure 1:**
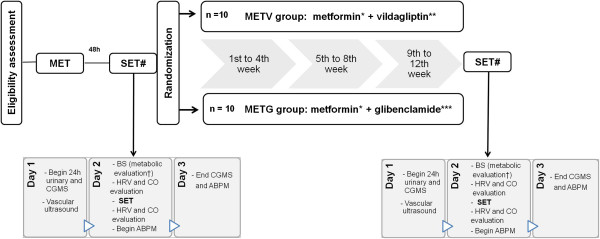
**Flow diagram of the study design.** # The submaximal exercise test (SET) will occur on the day 2 of a 3-day period of tests. METV group: 12 week s of treatment with vildagliptin added to metformin; METG group: 12 weeks of treatment with glibenclamide added to metformin. *500 to 2000 mg/day according to tolerance; **50 mg twice daily; ***10 to 20 mg twice daily. ABPM: ambulatory blood pressure monitoring, CO: cardiac output, CGMS: continuous glucose monitoring system; BS: blood sample; HRV: heart rate variability, MET: maximal exercise test. †Metabolic evaluation: glucose, HbA1c, insulin, glucagon-like peptide 1 (GLP-1).

The experimental sessions will occur at the Exercise Pathophysiology Research Laboratory, Hospital de Clínicas de Porto Alegre (LaFiEx-HCPA), maintaining the ambient temperature at between 20 and 22°C.Patients will be asked to follow their habitual diet during the 3 days of protocol. To carry out the submaximal exercise test they will go to LaFiEx-HCPA in a fasted state. Patients will record detailed food intake in diaries across the 3 days of CGMS use. The dietary assessment will be performed using the software DietWin (DietWin, Porto Alegre, Brazil). In addition, participants will be instructed to avoid intense activities and not to consume caffeinated beverages on the day before the dietary assessment, in order to exclude any residual effect before and after the effort test. The submaximal exercise test will occur after a standard breakfast (500 kcal; 60% carbohydrate, 30% fat and 10% protein). During the drug treatment period (12 weeks) patients will not follow any kind of physical exercise program. The flow diagram of the study design is shown in Figure 1.

### Study intervention

The METV group will receive 50 mg of vildagliptin orally twice a day. The METG group will receive glibenclamide 5 mg orally once per day during the first week of the study, then in the second week, the dose will be increased to 10 mg a day (5 mg twice a day). The dose may be adjusted to reach a target pre-prandial capillary plasma glucose level of 70 to 130 mg/dl without frequent or severe hypoglycemia, up to the maximum dose allowed (20 mg a day). Patients will be instructed to measure capillary glycemia using a glucose monitor (Accu-Check Performa; Roche Diagnostics, Mannheim, Germany) twice a week and at any time if they believe they have symptoms of hypoglycemia. These measured values will be recorded at the follow-up visits or passed on to the researchers in a phone call when two consecutive values lower than 70 mg/dl occur. This procedure will be explained at the beginning of the study. Medication adjustments will be guided by the researcher coordinator (BDS). Drug accountability will be assessed at each follow-up visit.

### Maximal exercise and submaximal exercise tests

A maximal exercise test will be performed to determine peak oxygen consumption (VO_2peak_) and ventilatory thresholds. Exercise capacity will be defined by a progressive maximal exercise test performed on a cycle ergometer, with increments of 20 W/min. The test will be carried out at 60 rpm until exhaustion. Oxygen consumption (VO_2_) and carbon dioxide production (VCO_2_) will be determined by averaging the gas exchange by a computerized system (Oxycon Delta; VIASYS Healthcare GmbH, Jaeger, Germany). The test will be finished when the individual is unable to maintain the 60 rpm speed. The VO_2_ peak is defined as the VO_2_ peak reached at the end of this exercise [13]. Heart rate will be continuously monitored by a 12-lead electrocardiogram (Nihon Kohden Corp., Tokyo, Japan).

The ventilatory thresholds will be determined at the breakpoint between the highest point of the CO_2_ production curve and VO_2_ (V slope), or at the point where the curves of ventilatory equivalent for oxygen (VE/VO_2_) and end tidal oxygen (PETO_2_) reach their respective minimum values and begin to increase. The respiratory compensation point will be determined when the levels of VE/VO_2_ reach their minimum values before they start to rise, and when PETCO_2_ reach their maximum values before these begin to decline [14].

The submaximal exercise tests are designed to simulate a typical aerobic exercise session on a cycle ergometer. The test will last 30 minutes, with an intensity based at 10% under the heart rate at the second threshold, which will be obtained in advance. Each patient will undergo two submaximal exercise tests, which will be performed before and after intervention. Before the test, at 15 minutes, at the end (30 minutes) and 60 minutes after each submaximal exercise test, 20 ml of blood will be collected and the variables described later will be measured, with the exception of HbA1c (measured just before exercise). The cardiovascular responses will be obtained as described below.

### Glucose variability evaluation

CGMS will be utilized for this evaluation. Subjects will be admitted to the laboratory in the morning at approximately 08.00 hours, 24 hours before the exercise session, when the glucose sensor (Sof-SensorTM; Medtronic Mini-Med Inc., Northridge, CA, USA) will be inserted subcutaneously. The sensor is a glucose oxidase-based platinum electrode that is inserted through a needle into the subcutaneous tissue of the anterior abdominal wall, using a spring-loaded device (Senserter, Medtronic Mini-Med Inc.).

Glucose oxidase catalyzes the oxidation of glucose in the interstitial fluid, which generates an electrical current. The current is carried by a cable to a pager-sized monitor that analyzes the data every 10 seconds and reports average values every 5 minutes, to give a total of 288 readings per day. Glucose profiles will be collected on the day before (day 1), the day of (day 2), and the day after (day 3) the submaximal exercise test. Sensor readings will be calibrated with a glucose monitor (Accu-Check Performa; Roche Diagnostics) using four finger-stick blood samples for each 24 hours. Each sensor will be used continuously for up to 48 hours. All patients will have been instructed previously about the operation of the monitor, which includes event registration for meals, and inserting capillary glucose values for calibration.

Glucose variability will be assessed from a series of absolute values of glucose, obtained by the CGMS, sampled every 5 minutes. Each method will be used as reported in the literature and according to its limitations. Glucose variability will be evaluated using conventional analysis and other mathematical methods generally applied to biological series, here called “non-conventional analysis of glucose variability.” Conventional analysis of glucose variability will be constructed from the statistical properties of the series, to obtain the following indices: MAGE [6], glucose variance (VAR), glucose coefficient of variation (CV%), and glucose SD, all normalized by the mean blood glucose in each period [15, 16]. These indices, except MAGE, will be calculated for every 6 hour block of glucose values to obtain the measures according to the specific period of the day. The MAGE index will be calculated for the whole signal (24 hours), and its calculation is based on the differences between consecutive points, considering those which are higher than 1SD [16].

Non-conventional analysis of glucose variability will be conducted using two methods applied to the glucose series: a linear method based on spectral analysis, and an integrated non-linear approach to the complexity analysis, called symbolic analysis. Spectral analysis is a linear method that allows quantification of the oscillatory components from time series by autoregressive models widely applied for heart rate and arterial pressure series [17]. Symbolic dynamics relies on the calculation of Shannon entropy of the distribution of patterns lasting three measures and the classification of frequent deterministic patterns lasting three measures, and distributes deterministic patterns of the group into four categories according to the number and pattern: 1) no variation (0 V), 2) one variation (1 V), 3) two like variations (2 LV), and 4) two unlike variations (2 UV) [18]. This method has been fully described and validated previously to glucose curves [7].

### Metabolic evaluation

Peripheral venous blood will be collected into 10 ml vacutainer tubes, and used to perform the blood tests; these samples will be stored at −20°C. Glucose, HbA1c, insulin, glucagon, and glucagon-like peptide 1 (GLP-1) will be assayed.

### Oxidative stress evaluation

The 24-hour urinary samples will be collected at visits 1 and 6 and used to evaluate creatinine and 8-isoprostaglandin F2α (8-isoPGF2α), which is considered a well-recognized marker of oxidative stress [19]. This isomer will be measured using a competitive enzyme-linked immunoassay (ELISA) (Cell Biolabs Inc. San Diego, CA USA).

### Cardiovascular evaluation

Cardiac output and heart rate will be measured before (10 minutes at rest) and during the submaximal exercise test and after 60 minutes of recovery, using a non-invasive method. The data will be recorded (MP150 system; Biopac Santa Barbara, CA, USA) using a general purpose amplifier module (DA100; Biopac), impedance cardiography (NICO100C; Biopac) and electrocardiogram (ECG100C; Biopac), according to methodological guidelines provided by Sherwood *et al*. [20].

To assess the cardiovascular autonomic control, heart rate variability (HRV) analysis will be applied. Pulse intervals (PI) series (tachograms) will be obtained from the electrocardiography records. Stationary segments (300 beats), coincident in the tachogram, will be selected, and spectral analysis will be performed using an autoregressive model, which estimates the center frequency and power of each relevant oscillatory component. The spectral bands for humans are defined as very low frequency (VLF), for 0.0 to 0.04 Hz, low frequency (LF), for 0.04 to 0.15 Hz, and high frequency (HF) for 0.15 to 0.40 Hz intervals, defined according to previous references [17]. Tachogram spectra will be evaluated quantitatively, and values of HRV will be obtained, as well as its spectral components, and will be expressed in absolute (ms2). In additional, LF and HF bands will be expressed in normalized units (NU), obtained by calculating the power of LF and HF spectra, and correlating them with the total power without the VLF component [17].

Of the parameters that can be obtained by spectral analysis, those distinguished for their physiological significance are the LF and HF components, which are mainly related to sympathetic and parasympathetic cardiac modulations, respectively. The relationship between them – the LF/HF index, or sympathetic-vagal balance [21] – will be evaluated.

Patients will be submitted to a 24 hour ABPM on a usual work day, using a monitor (model 90207; Spacelabs, Redmond, WA, USA) that will be programmed to automatically measure BP every 15 minutes during the day (06.00 to 22.00 hours), and every 20 minutes during the night (22.00 to 6.00 hours) [22, 23]. BP variability will be assessed from BP behavior in different windows of a 24 hour period, covering both daytime and night-time periods. The cuff size will be adapted to the circumference of the arm of each patient according to the manufacturer’s recommendations.

Based on the results of the 24 hour ABPM, the mean 24 hour systolic BP (SBP) and diastolic BP (DBP) will be calculated for each patient, before and after treatment. Three different parameters of SBP variability will be calculated: 1) time–rate index (rate of change in SBP over time in mm Hg/min, defined as the first derivative values of SBP by time); 2) coefficient of variation of the 24 hour SBP (SD/mean pressure × 100%); and 3) mean of the SD of the 24 hour SBP. The time–rate index allows the calculation of the sum of angular coefficients, and aims to measure how fast or how slow SBP values are and in which direction they change. The measure will be calculated using the formula,


where *r* is the rate of BP variability over time (considering the differences between BP measurements at each time interval) and *N* is the number of recordings [24]:

### Endothelial function evaluation

This analysis will be performed by a high-resolution ultrasound of the brachial artery (vascular Doppler), which characterizes the flow-mediated dilatation (FMD). FMD is expressed by changes in basal diameter in response to the increased flow and to nitroglycerin, which will be administered in a single dose (0.4 mg, sublingual spray). The equipment used will be an HD7XE (Philips, Bothell, WA, USA) with an HF transducer (3 to 12 MHz; L12-3; Philips) [25].

### Ethics and data protection issues

Participation will be voluntary, and will follow the ethical aspects of confidentiality and data protection. Procedures will be explained to patients, and information about the aim, design, associated potential risks and benefits and all relevant details of the research will be given in the informed consent form. All patients will have to sign the informed consent form prior to participating in the study. The data obtained by the study will be available to the participant and to any other authorized person, and may be used anonymously for academic scientific purposes.

The study was approved by Scientific Committee and Research Ethical Comission (IRB number 120444) of Hospital de Clínicas de Porto Alegre (Brazil), which is part of National Committee of Ethics in Research. Approval Number: 10662912.3.0000.5327.

### Safety assessment

Adverse events (AEs), including serious adverse events (SAEs) or pregnancies will be collected and included in the medical reports. The reports containing details of SAEs or pregnancies will be forwarded to the laboratory of the respective manufacturer within 24 hours, and to the health authorities.

To ensure patient safety, the individuals participating in the research will be monitored for the occurrence of all AEs after beginning the specific protocol procedures until 4 weeks after the patient discontinues participation in the study.

### Statistical analysis plan

Data will be analyzed using SPSS software (Statistical Package for Social Sciences; version 18.0 for Windows, SPSS Inc., Chicago, USA). The description of values will be expressed as mean ± SD. The statistical procedures used will be Student’s *t*-test for independent samples (intergroup analysis), paired *t*-test (intragroup analysis), and analysis of variance (ANOVA) for repeated measures to compare both groups at different times. A Pearson correlation will be performed between measures of glucose variability and 8-isoPGF2α/crn. The statistical power will be 90%, and the accepted level of significance will be *P* < 0.01.

## Discussion

Several anti-diabetic agents are available for T2DM treatment. These treatments differ in their mechanism of drug action and their long-term outcomes. In addition to these, non-pharmacological treatments are important, especially exercise, as it is a major tool to achieve target blood glucose and HbA1c. Non-glucose beneficial effects can also be obtained. The many studies reporting the effects of the association between exercise and oral glucose-lowering drugs usually focus on attaining better metabolic control. This study will be conducted to report the glucose variability and cardiovascular responses at rest, during and after submaximal exercise under two oral glucose-lowering drugs (vildagliptin versus glibenclamide).

## Trial status

Not yet recruiting. Enrolment will begin in April 2014. Each patient will have 10 visits to the hospital, and total data collection time will be 16 months.

## Authors’ information

AF is a PhD student in the Postgraduate Program of Cardiology, School of Medicine, Universidade Federal do Rio Grande do Sul, who performs investigations in the Exercise Pathophysiology Research Laboratory – Hospital de Clínicas de Porto Alegre.

JPR worked in the Cardiology Division, Hospital de Clínicas de Porto Alegre, RS, Brazil, and was a professor in Postgraduate Program of Cardiology, School of Medicine, Universidade Federal do Rio Grande do Sul.

KRC is a professor in the Department of Science and Technology, Science and Technology Institute, Universidade Federal de São Paulo, São José dos Campos, Sao Paulo, Brazil.

BDS works in the Endocrine Division, Hospital de Clínicas de Porto Alegre, RS, Brazil, and is a professor in Postgraduate Program of Endocrinology, School of Medicine, Universidade Federal do Rio Grande do Sul.

## Abbreviations

8-iso PGF2 α: 8-iso prostaglandin F2α
ABPM: Ambulatory blood pressure monitoring
BMI: Body mass index
BP: Blood pressure
CGMS: Continuous glucose monitoring system
CV%: Glucose coefficient of variation
DBP: diastolic blood pressure
FMD: Flow-mediated dilatation
GLP-1: Glucagon-like peptide 1
HbA1c: Glycated hemoglobin
HF: High frequency
HRV: Heart rate variability
LF: Low frequency
MAGE: Mean amplitude of glycemic excursion
METG: Metformin plus glibenclamide group
METV: Metformin plus vildagliptin group
NU: Normalized units
PETCO_2_: Tidal carbon dioxide
PETO_2_: End tidal oxygen
PI: Pulse interval
SBP: Systolic blood pressure
SD: Standard deviation
T2DM: Patients with type 2 diabetes
VAR: Glucose variance
VCO_2_: Carbon dioxide production
VE/VO_2_: Ventilatory equivalent for oxygen
VLF: Very low frequency
VO_2_: Oxygen consumption
VO_2peak_: Peak oxygen consumption.

## References

[1] Bloomgarden ZT (2010). Cardiovascular disease in diabetes. Diabetes Care.

[2] Natali A, Ferrannini E (2012). Endothelial dysfunction in type 2 diabetes. Diabetologia.

[3] Kuenen JC, Borg R, Kuik DJ, Zheng H, Schoenfeld D, Diamant M, Nathan DM, Heine RJ, Group AS (2011). Does glucose variability influence the relationship between mean plasma glucose and HbA1c levels in type 1 and type 2 diabetic patients?. Diabetes Care.

[4] Monnier L, Mas E, Ginet C, Michel F, Villon L, Cristol JP, Colette C (2006). Activation of oxidative stress by acute glucose fluctuations compared with sustained chronic hyperglycemia in patients with type 2 diabetes. JAMA.

[5] Di Flaviani A, Picconi F, Di Stefano P, Giordani I, Malandrucco I, Maggio P, Palazzo P, Sgreccia F, Peraldo C, Farina F, Frajese G, Frontoni S (2011). Impact of glycemic and blood pressure variability on surrogate measures of cardiovascular outcomes in type 2 diabetic patients. Diabetes Care.

[6] Service FJ, Molnar GD, Rosevear JW, Ackerman E, Gatewood LC, Taylor WF (1970). Mean amplitude of glycemic excursions, a measure of diabetic instability. Diabetes.

[7] Figueira FR, Umpierre D, Casali KR, Tetelbom PS, Henn NT, Ribeiro JP, Schaan BD (2013). Aerobic and combined exercise sessions reduce glucose variability in type 2 diabetes: crossover randomized trial. PLoS One.

[8] Marfella R, Barbieri M, Grella R, Rizzo MR, Nicoletti GF, Paolisso G (2010). Effects of vildagliptin twice daily vs. sitagliptin once daily on 24-hour acute glucose fluctuations. J Diabetes Complications.

[9] Umpierre D, Ribeiro PA, Kramer CK, Leitão CB, Zucatti AT, Azevedo MJ, Gross JL, Ribeiro JP, Schaan BD (2011). Physical activity advice only or structured exercise training and association with HbA1c levels in type 2 diabetes: a systematic review and meta-analysis. JAMA.

[10] Lin SD, Wang JS, Hsu SR, Sheu WH, Tu ST, Lee IT, Su SL, Lin SY, Wang SY, Hsieh MC (2011). The beneficial effect of α-glucosidase inhibitor on glucose variability compared with sulfonylurea in Taiwanese type 2 diabetic patients inadequately controlled with metformin: preliminary data. J Diabetes Complications.

[11] Gibbons RJ, Balady GJ, Bricker JT, Chaitman BR, Fletcher GF, Froelicher VF, Mark DB, McCallister BD, Mooss AN, O’Reilly MG, Winters WL, Gibbons RJ, Antman EM, Alpert JS, Faxon DP, Fuster V, Gregoratos G, Hiratzka LF, Jacobs AK, Russell RO, Smith SC (2002). ACC/AHA 2002 guideline update for exercise testing: summary article: a report of the American College of Cardiology/American Heart Association Task Force on Practice Guidelines (Committee to Update the 1997 Exercise Testing Guidelines). Circulation.

[12] Heald CL, Fowkes FG, Murray GD, Price JF, Collaboration ABI (2006). Risk of mortality and cardiovascular disease associated with the ankle-brachial index: Systematic review. Atherosclerosis.

[13] Foss Ø, Hallén J (2005). Validity and stability of a computerized metabolic system with mixing chamber. Int J Sports Med.

[14] Wasserman K, Whipp BJ, Koyl SN, Beaver WL (1973). Anaerobic threshold and respiratory gas exchange during exercise. J Appl Physiol.

[15] Zaccardi F, Stefano PD, Busetto E, Federici MO, Manto A, Infusino F, Lanza GA, Pitocco D, Ghirlanda G (2008). Group of signs: a new method to evaluate glycemic variability. J Diabetes Sci Technol.

[16] Hill NR, Oliver NS, Choudhary P, Levy JC, Hindmarsh P, Matthews DR (2011). Normal reference range for mean tissue glucose and glycemic variability derived from continuous glucose monitoring for subjects without diabetes in different ethnic groups. Diabetes Technol Ther.

[17] Malliani A, Pagani M, Lombardi F, Cerutti S (1991). Cardiovascular neural regulation explored in the frequency domain. Circulation.

[18] Porta A, Guzzetti S, Montano N, Furlan R, Pagani M, Malliani A, Cerutti S (2001). Entropy, entropy rate, and pattern classification as tools to typify complexity in short heart period variability series. IEEE Trans Biomed Eng.

[19] Morrow JD, Hill KE, Burk RF, Nammour TM, Badr KF, Roberts LJ (1990). A series of prostaglandin F2-like compounds are produced in vivo in humans by a non-cyclooxygenase, free radical-catalyzed mechanism. Proc Natl Acad Sci U S A.

[20] Sherwood A, Allen MT, Fahrenberg J, Kelsey RM, Lovallo WR, van Doornen LJ (1990). Methodological guidelines for impedance cardiography. Psychophysiology.

[21] Montano N, Porta A, Cogliati C, Costantino G, Tobaldini E, Casali KR, Iellamo F (2009). Heart rate variability explored in the frequency domain: a tool to investigate the link between heart and behavior. Neurosci Biobehav Rev.

[22] O'Brien E, Parati G, Stergiou G, Asmar R, Beilin L, Bilo G, Clement D, de la Sierra A, de Leeuw P, Dolan E, Fagard R, Graves J, Head GA, Imai Y, Kario K, Lurbe E, Mallion JM, Mancia G, Mengden T, Myers M, Ogedegbe G, Ohkubo T, Omboni S, Palatini P, Redon J, Ruilope LM, Shennan A, Staessen JA, van Montfrans G, Verdecchia P, Waeber B, Wang J, Zanchetti A, Zhang Y, European Society of Hypertension Working Group on Blood Pressure Monitoring (2013). European Society of Hypertension position paper on ambulatory blood pressure monitoring. J Hypertens.

[23] Hansen TW, Thijs L, Li Y, Boggia J, Kikuya M, Björklund-Bodegård K, Richart T, Ohkubo T, Jeppesen J, Torp-Pedersen C, Dolan E, Kuznetsova T, Stolarz-Skrzypek K, Tikhonoff V, Malyutina S, Casiglia E, Nikitin Y, Lind L, Sandoya E, Kawecka-Jaszcz K, Imai Y, Wang J, Ibsen H, O'Brien E, Staessen JA, International Database on Ambulatory Blood Pressure in Relation to Cardiovascular Outcomes Investigators (2010). Prognostic value of reading-to-reading blood pressure variability over 24 hours in 8938 subjects from 11 populations. Hypertension.

[24] Zakopoulos NA, Tsivgoulis G, Barlas G, Papamichael C, Spengos K, Manios E, Ikonomidis I, Kotsis V, Spiliopoulou I, Vemmos K, Mavrikakis M, Moulopoulos SD (2005). Time rate of blood pressure variation is associated with increased common carotid artery intima-media thickness. Hypertension.

[25] Corretti MC, Anderson TJ, Benjamin EJ, Celermajer D, Charbonneau F, Creager MA, Deanfield J, Drexler H, Gerhard-Herman M, Herrington D, Vallance P, Vita J, Vogel R, International Brachial Artery Reactivity Task Force (2002). Guidelines for the ultrasound assessment of endothelial-dependent flow-mediated vasodilation of the brachial artery: a report of the International Brachial Artery Reactivity Task Force. J Am Coll Cardiol.

